# Fatal Rhinofacial Mycosis Due to *Aspergillus nomiae*: Case Report and Review of Published Literature

**DOI:** 10.3389/fmicb.2020.595375

**Published:** 2020-12-22

**Authors:** Ya Bin Zhou, Dong Ming Li, Jos Houbraken, Ting Ting Sun, G. Sybren de Hoog

**Affiliations:** ^1^Mycological Laboratory, Department of Dermatology, Peking University Third Hospital, Beijing, China; ^2^Westerdijk Fungal Biodiversity Institute, Utrecht, Netherlands; ^3^Center of Expertise in Mycology, Radboud University Medical Center/Canisius Wilhelmina Hospital, Nijmegen, Netherlands

**Keywords:** *Aspergillus nomiae*, rhinofacial mycosis, skin and soft tissue infections, leukemia, rhino-orbital-cerebral mucormycosis, antifungal resistance

## Abstract

**Background:**

A 73-year-old female suffering from acute myeloid leukemia presented with progressive rhinofacial mycosis. Suspecting it to be mucormycosis, the antifungal amphotericin B (AMB) was administered empirically, but the patient did not respond as planned. The fungus was then isolated from the biopsied tissue and morphologically identified as a species of *Aspergillus*. Necrosis progressed and she died of cerebral hemorrhage. Since *Aspergillus flavus* is susceptible to AMB, and several other *Aspergillus* species can be misidentified as *A. flavus*, the observed resistance necessitated a re-examination of the fungal isolate.

**Methods:**

The fungal strain was re-isolated and re-examined morphologically. Additionally, genomic DNA was extracted from the fungus and sequences were obtained from three genomic regions [the rDNA internal transcribed spacer (ITS) region, and portions of the β-tubulin and calmodulin genes] to more accurately identify this *Aspergillus* strain. Its antifungal susceptibility was assessed using multiple compounds and our findings were compared with literature data.

**Results:**

The fungal culture again yielded an *Aspergillus* isolate morphologically identical to *A. flavus*. Molecular analyses, however, revealed the strain to be *A. nomiae*, a close relative of *A. flavus* in section *Flavi*, and it exhibited resistance to AMB. Reviewing the literature, only five other cases of *A. nomiae* infection in humans have been reported worldwide.

**Conclusion and Clinical Importance:**

The rhinofacial mycosis of the patient was actually due to *A. nomiae*. The initial misidentification of the fungus, coupled with its resistance to AMB, could be the reason treatment did not help the patient. We postulate that clinical *A. nomiae* infections may be underreported and that accurate and speedy pathogen identification is important so that an effective antifungal regimen can be administered.

## Introduction

Invasive aspergillosis, i.e., deep infections by an *Aspergillus* species, are among the most common opportunistic mold infections that can cause potentially life-threatening disease in those with chronic neutropenia, or inherited or acquired immunodeficiencies, as well as in those undergoing allogeneic hematopoietic stem cell transplant, solid organ transplant, or prolonged corticosteroid use (Li et al., 2008; Patterson et al., 2016). Among these, pulmonary infection and rhino-(facial)-orbital-cerebral mycosis (ROCM) are the most problematic types (Patterson et al., 2016; Leroy et al., 2020; Hu et al., 2021). Second only to *Aspergillus fumigatus* for lung infections and ROCM (Alsalman et al., 2017; Rudramurthy and Paul, 2019; Hu et al., 2021), *Aspergillus flavus* is the most frequently encountered species in invasive sinusitis (Bakhshaee et al., 2016; Alsalman et al., 2017); in 9% of cases of ROCM, *A. flavus* is listed as the causal organism (Hu et al., 2021), while other species have remained unidentified in the literature (Hu et al., 2021). Although clinical outcomes of treatment of aspergilloses have markedly improved with the availability of newer triazoles, the development of resistance to triazoles in *A. fumigatus* and the intrinsic resistance to polyenes in *A. flavus* is a growing concern (Rudramurthy and Paul, 2019; Wiederhold and Verweij, 2020). The understanding of antifungal drug resistance at the species level is of great significance for clinical selection of empirical drug therapy.

*Aspergillus flavus* has a number of similar molecular siblings. Phylogenetically, the *Aspergillus* section *Flavi* is split into eight series: *Alliacei*, *Avenacei*, *Bertholletiarum*, *Coremiiformes*, *Flavi*, *Kitamyces*, *Leporum*, and *Nomiarum* (Frisvad et al., 2019; Houbraken et al., 2020). *A. flavus* can have two morphotypes (L and S) based on sclerotium production; significant morphological, physiological, and pathological differences exist between the two morphotypes (Gilbert et al., 2018). Given that *A. flavus* shares a similar morphology with other closely related species, potential misidentification of an isolate as *A. flavus* is an issue. Molecular investigations of certain conserved loci (e.g., ITS, *BenA*, *CaM*) can help in determining the species. With high morphological similarities, conserved loci most often help to delineate species of fungi (Houbraken et al., 2020).

*Aspergillus* strains can produce B-series and G-series aflatoxins (Ehrlich et al., 2004), which are notorious for hepatocellular carcinoma development, lung adenocarcinoma, and chronic inflammatory changes. Infection by aflatoxin-producing species was reported in patients with leukemia (Mori et al., 1998; Cha and Dematteo, 2005; Liu et al., 2015). Being a new aflatoxin-producing species belonging to *Aspergillus* section *Flavi*, *Aspergillus nomiae* strains are able to produce both series B and G aflatoxins (Ehrlich et al., 2004). However, the relationship between aflatoxin-producing *Aspergillus* infection and leukocytosis or leukemia is unclear (Mori et al., 1998).

Rhinofacial fungal infections are almost entirely restricted to entomophthoromycoses and mucormycosis (Li and Lun, 2012; Li et al., 2014; Shaikh et al., 2016), very specific clinical entities that are usually susceptible to the antifungal compound amphotericin B (AMB) (Li et al., 2014). Here, we report an erosive case of invasive rhinofacial fungal infection that was resistant to antifungal treatment with AMB and contributed to the death of the patient after only 7 days in our hospital. *A. flavus* has been reported to be variably resistant to AMB (Hagiwara et al., 2016; Jalalian and Shokohi, 2020), the most commonly used antifungal in cases of severe infection in high-risk patients (Li and de Hoog, 2009; Li and Lun, 2012; Li et al., 2014). Antifungal resistance may be species-dependent, therefore rapid and accurate identification of resistant species is of great importance. The present case report of rhinofacial fungal infection concerns a fungus that was resistant to AMB. This resistant fungus warranted further study, which included both morphological and molecular investigations, supplemented with a literature search into previous clinical cases of fungal resistance to AMB. In this study, we revisited the original case file and determined the need to re-examine the fungal isolate using morphological and molecular techniques, to assess its antifungal susceptibility profile, and (once accurately identified) to search the literature for other cases involving antifungal resistance by this particular *Aspergillus* species in a clinical setting.

## Materials and Methods

### Case Report

In November of 2014, a 73-year-old female with acute myeloid leukemia presented to Peking University Third Hospital (Beijing, China) with progressive nose swelling and ulceration after a severe cold. The lesions had begun two months earlier as infiltrated erythema, then rapidly increased in size and ulcerated. The patient was initially diagnosed with skin and subcutaneous infection in the nose and face (rhinofacial) and treatment with antibiotics was ineffective. Next, the lesion was diagnosed as a malignant ulcer and chemotherapy was administered, but the necrotic ulcer became progressively enlarged. Subsequent clinical diagnosis of rhinofacial mucormycosis was made and the patient was treated with AMB (initial dose 5 mg/day, increased by 5 mg daily), but the ulcer still progressed. Two months after onset of the malignant ulcer, the patient was transferred to Peking University Third Hospital.

**FIGURE 1:**
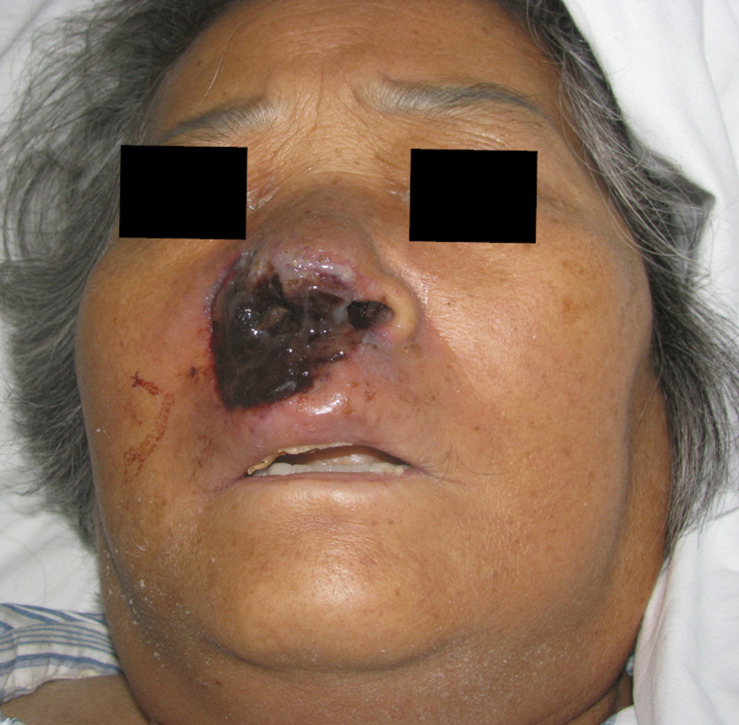
Image of patient exhibiting edematous erythema, ulcer, and necrosis over the right nasal alae and upper lip.

The patient had a 1-year history of acute myeloid leukemia and had received one cycle of DA chemotherapy (daunorubicin and cytarabine), two cycles of MA chemotherapy (mitoxantrone and cytarabine), one cycle of CAG chemotherapy (cytarabine, aclarubicin, and recombinant human granulocyte-colony stimulating factor), and one cycle of AA chemotherapy (aclarubicin and cytarabine). She denied any history of preceding injury or insect bite at the site of infection. Physical examination revealed erythema, edema, and ulceration over the right nasal alae and upper lip, extending to the soft tissue around the right side of the nose (Figure 1).

**FIGURE 2:**
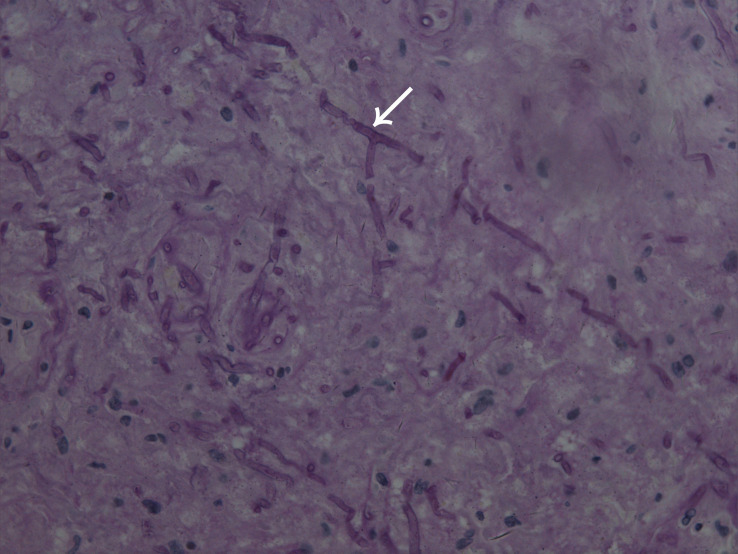
Microscope image of biopsied lesion tissue showing numerous hyaline and septate hyphae with branches at acute angles scattered in the dermis (arrowhead) (periodic acid–Schiff, original magnification × 400).

**FIGURE 3:**
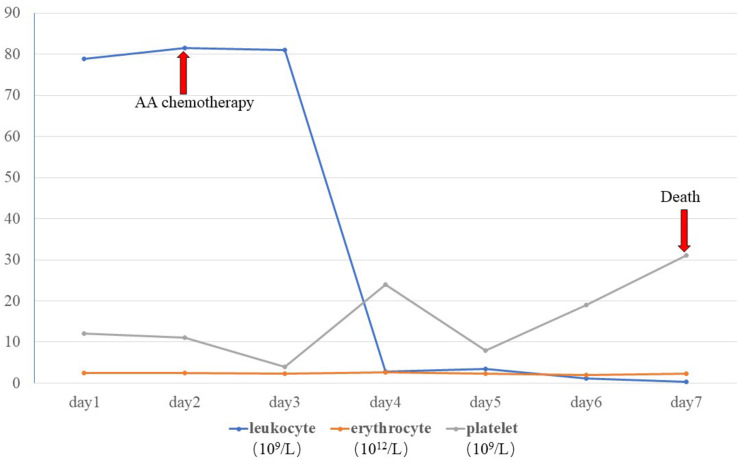
Blood cell counts during hospitalization.

A skin biopsy was performed on the lesion of the nasal dorsum. Histopathological examination later revealed inflammation, necrosis, granuloma, and angiodestruction. In addition, numerous hyaline and septate hyphae with branches at acute angles were observed throughout the dermis with angioinvasion (Figure 2). The laboratory evaluation revealed hyperleukocytosis (78.9 × 10^9^/L), erythrocytopenia (2.44 × 10^12^/L), thrombocytopenia (12 × 10^9^/L), hypokalemia (2.38 mmol/L), an elevated level of C-reactive protein (31.2 mg/dL), and a positive serum galactomannan (GM) test (1.25 μg/L). The results of the antinuclear antibody test, T cell enzyme-linked immunospot assay, and serum (1-3)-β-d-glucan (G) test were all negative. Serum hepatitis B surface antigen and antibodies to human immunodeficiency virus, hepatitis C virus, and *Treponema pallidum* were all negative. Leukocyte counts decreased rapidly after receiving AA chemotherapy during hospitalization (Figure 3).

Despite 7 days of continuous treatment with AMB (before biopsy), the ulcer progressed and the patient died of cerebral hemorrhage. The biopsied tissue yielded an *Aspergillus* isolate that was initially identified by morphology as *A. flavus.* However, the antifungal susceptibility profile of the culture showed resistance to AMB.

### Re-assessment of Fungal Morphology

The biopsied specimen was originally cultured and stored on Sabouraud’s glucose agar (SGA) medium in the Peking University Third Hospital collection under accession number PUTH 1342. For this study, we recovered the fungus from the original culture by inoculation onto potato dextrose agar (PDA) media and incubation at 37°C for 7 days. We then conducted macro- and micro-morphological examinations to identify the fungal pathogen. On the basis of these microscopic and macroscopic characteristics, the fungus was identified as a member of the *Aspergillus* section *Flavi*.

### Molecular Investigations

Genomic DNA of the fungal isolate was extracted using the Biospin Fungus Genomic DNA Extraction kit (Bioer Technology, Hubei, China) in accordance with manufacturer’s instructions. The internal transcribed spacer (ITS) region was amplified using the ITS5 and ITS4 primers (Zhou et al., 2019), a segment of the β-tubulin gene (*BenA*) was amplified with βtub1 and βtub2 primers (Balajee et al., 2005), as well as a segment of the calmodulin gene (*CaM*) with cmd5 and cmd6 primers (Hong et al., 2005). Each PCR mixture contained 1 μL extracted fungal DNA, 0.08 μM each of the primers, and 12.5 μL 2 × Taq PCR MasterMix (Tiangen Biotech, Beijing, China) in a 25 μL reaction volume. PCR cycles comprised of an initial denaturation at 95°C for 5 min, followed by 35 cycles of 95°C for 30 s, 58°C for 30 s, and 72°C for 1 min, a final extension at 72°C for 10 min, and cooling to 4°C. The amplicons were then sequenced by BGI Company (Beijing, China). The sequences obtained were compared with those deposited in GenBank by BLAST query.

Based on BLAST findings, a phylogenetic analysis was performed based on concatenation of the three genomic regions (ITS, *BenA*, and *CaM*) to help determine the relationship between our isolate and seven reference type strains from section *Flavi*, as well as additional representatives of the identified species (Table 1). Maximum likelihood analysis of the individual datasets was performed using MEGA7 and the robustness of the trees was evaluated by 1000 bootstrap replicates.

**TABLE 1:** List of strains used in this study.

			**GenBank accession no.**		
**Species**	**Isolate/strain number**	**Substrate and location**	**ITS**	***BenA***	***CaM***
*A. alliaceus*	CBS 536.65^*T*^	Dead blister beetle (*Microbasis albida*), Washington, DC, United States, ex neotype of *A. alliaceus*	EF661551	EF661465	EF661534
*A. flavus*	NRRL 1957^*T*^ (=CBS 100927)	Cellophane diaphragm of an optical mask, South Pacific Islands, ex type of *A. flavus*	AF027863	EF661485	EF661508
*A. luteovirescens*	CBS 620.95^*T*^	Unknown source, ex type of *A. luteovirescens*	MG662406	MG517625	MG517998
*A. luteovirescens*	NRRL 25593	Frass in a sílkworm rearing house, Japan	AF104445	EF661497	EF661532
*A. minisclerotigenes*	CBS 117635^*T*^	*Arachis hypogea*, Manfredi, Córdoba province, Argentina, ex type of *A. minisclerotigenes*	EF409239	EF203148	MG518009
*A. nomiae*	CBS 399.93	Soil, Guandong, Zhaoqing, China	FJ491472	MG517757	MG518127
*A. nomiae*	DTO 161-F2	Bamboo sample, Addis Abeba, Ethiopia	MH279388	MG517657	MG518027
*A. nomiae*	DTO 247-F9	House dust, Mexico	*	MG517723	MG518093
*A. nomiae*	DTO 318-F4	Heat treated pectin, Germany	*	MG517761	MG518131
*A. nomiae*	MUT 4191 (=CBS 124572)	Onychomycosis, Italy (Zotti et al., 2011)	*	HM640219	-
*A. nomiae*	NRRL 13137^*T*^ (=CBS 260.88)	Wheat, United States, ex type of *A. nomius*	AF027860	EF661494	EF661531
*A. nomiae*	PUTH 1342	Acute myeloid leukemia, China (this study)	MK088520	MK105849	MK105850
*A. nomiae*	PW2955	Human bronchoalveolar lavage, China (Tam et al., 2014)	KF562198	KF562209	KF562220
*A. nomiae*	PW2959	Human bronchoalveolar lavage, China (Tam et al., 2014)	KF562202	KF562213	KF562224
*A. novoparasiticus*	CBS 126849^*T*^	Sputum of leukemia patient, Sao Paolo, Brazil, ex type of *A. novoparasiticus*	MG662397	MG517684	MG518055
*A. pseudonomiae*	823/07	Human corneal infiltrate, India (Manikandan et al., 2009)	GQ221261	GQ221262	GQ221263
*A. pseudonomiae*	DTO 267-D6	House dust, Micronesia	MH279416	MG517731	MG518101
*A. pseudonomiae*	DTO 267-H7	House dust, Thailand	MH279417	MG517732	MG518102
*A. pseudonomiae*	NRRL 3353^*T*^ (=CBS 119388)	Diseased alkali bee (*Nomiae* sp.), Wyoming, United States; ex type of *A. pseudonomiae*	AF338643	EF661495	EF661529

*Sequences were not included in the phylogenetic analysis.

### Antifungal Susceptibility Testing

Antifungal susceptibility testing was performed using the CLSI M28-A2 microbroth dilution method. Minimal inhibitory concentrations (MIC) and minimum effective concentrations (MEC) were determined after growth at 35°C for 46–50 h for all antifungal agents tested. The MIC endpoints for itraconazole, voriconazole, posaconazole, and AMB were determined using a reading mirror as the lowest concentration of the drug that prevented any recognizable growth (100% inhibition). The MIC results were interpreted according to the revised clinical breakpoints v. 10.0 of the European Committee on Antimicrobial Susceptibility Testing (EUCAST), who defines new criteria for resistance to AMB (S ≤ / > R = 1/1) (Arendrup et al., 2020). The other revised breakpoints include itraconazole (ATU = 2) and isavuconazole against *A. flavus* (S usvuszolevucoATU = 2); isavuconazole (S ≤ / > R = 1/2, ATU = 2), itraconazole [S conazole (S oATU = 2)], posaconazole (ATU = 0.25), and voriconazole [S conazole (S iATU = 2)] against *A. fumigatus*, itraconazole (S conazoletracoATU = 2), and voriconazole [S conazole (S nATU = 2)] against *Aspergillus nidulans*, AMB against *Aspergillus niger* (S rgerst n B) against itraconazole [S conazole (S gATU = 2)], and posaconazole (ATU = 0.25) against *Aspergillus terreus*. MEC endpoints for caspofungin and micafungin were defined microscopically as the lowest concentration of drug that led to the growth of small, rounded, compact hyphal forms, compared to the long, unbranched hyphal clusters observed in the growth control. *Candida krusei* ATCC 6258, *Candida parapsilosis* ATCC 22019, and *A. flavus* ATCC 204304 were used as quality controls.

### Literature Search

After the species identity of our causal fungus was confirmed, an electronic search was performed using the correct species name and “human infection” in the PubMed database^1^.

## Results

**FIGURE 4:**
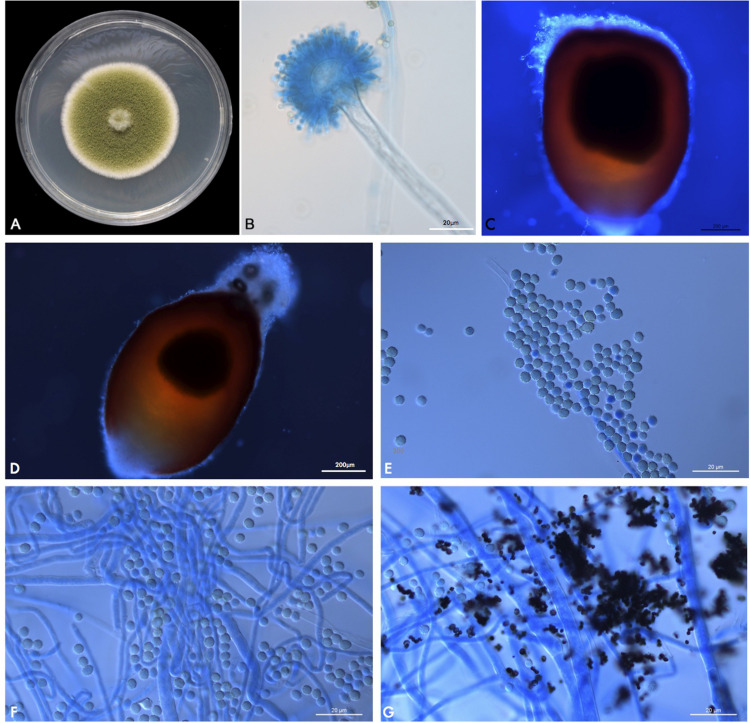
**(A)** PUTH 1342: Macromorphology on PDA at 37°C after 7 days of incubation. **(B)** Microscopic appearance of PUTH 1342 conidiophore (lactophenol cotton blue, original magnification × 400). **(C,D)** Bullet-shaped sclerotia. **(E)** Rough-walled subspherical conidia, measuring 2–5 μm diameter. **(F,G)** Hyaline and septate hyphae with branches at acute angles.

A velvety, yellow-green colony was observed on PDA (Figure 4A) after 1 week at 25°C. After prolonged incubation (>2 weeks), bullet-shaped sclerotia appeared. Microscopic examination of the fungus revealed hyaline and septate hyphae with branches at acute angles. Conidiophores had roughened stipes measuring 400–1000 μm; conidiophore heads had a spherical vesicle of 20–50 μm in diameter, fertile on more than three quarters of their surface, and carrying both metulae and phialides of 7–10 μm long. Conidia were rough-walled, subspherical, and measuring 2–5 μm diameter (Figures 4B–G). This morphology matched the description of the fungus given by de Hoog et al. (2019).

BLAST query of all three genomic regions indicated PUTH 1342 was actually *Aspergillus nomius*, which has recently been renamed as *A. nomiae*. These sequences have been deposited in GenBank under respective accession numbers MK088520, MK105849, and MK105850.

**FIGURE 5:**
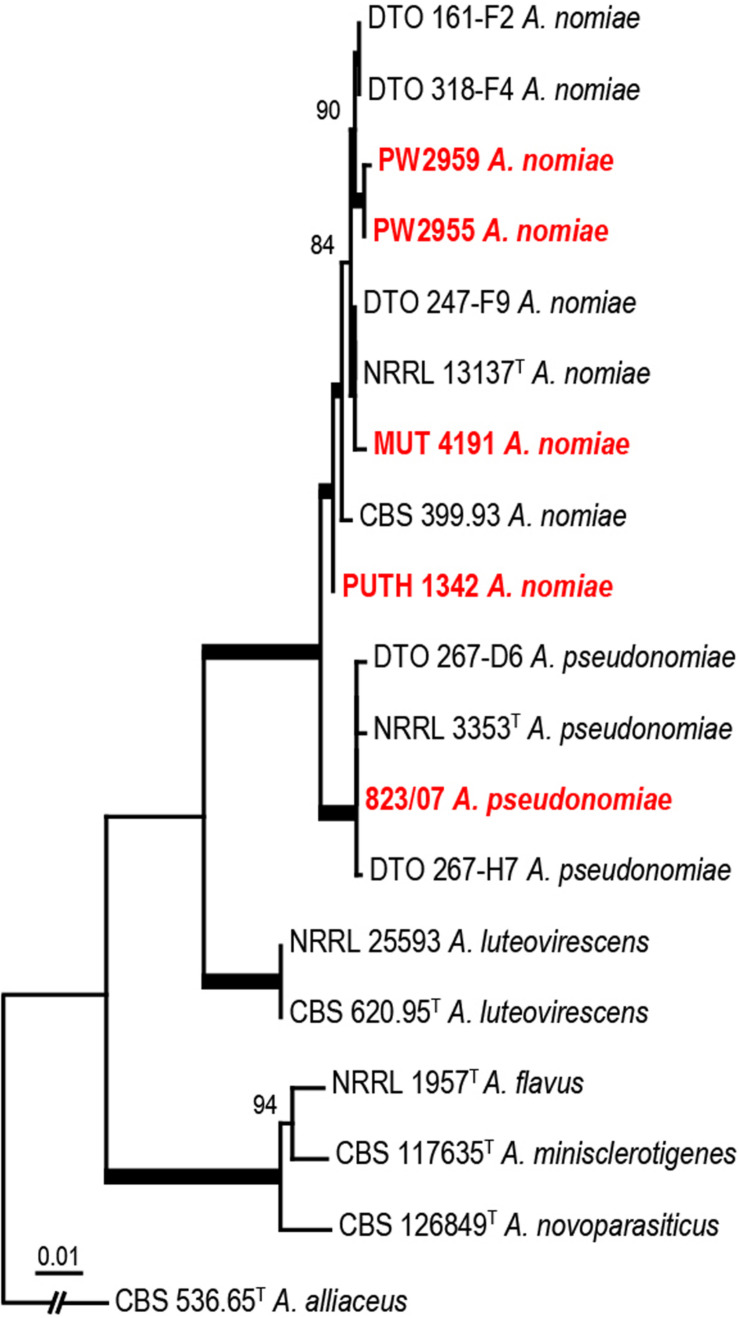
Phylogenetic analysis of three concatenated genomic regions (ITS, *BenA*, and *CaM*) involving PUTH 1342, seven reference strains, and closely related species from section *Flavi*. *A. alliaceus* were used as outgroup. The *A. nomiae* and *A. pseudonomiae* isolates in red were previously isolated from clinical specimens. The phylogram was constructed by the maximum likelihood method with 1000 bootstrap replicates.

PUTH 1342 clustered most closely with the *A. nomiae* type strain (Figure 5) and formed a clade with other *A. nomiae* strains, including other clinical isolates that were identified as *A. nomius* in previous studies (PW 2955, PW 2959, and MUT 4191). PUTH 1342 was therefore confidently identified as *A. nomiae* (Table 2 and Figure 5) (Houbraken et al., 2020).

**TABLE 2:** Cases of *A. nomiae* infection of humans reported in the literature.

**Report year (references)**	**Patient age/gender**	**Country**	**Predisposing factors**	**Clinical Presentation**	**Treatment**	**Outcome**
2009 (Manikandan et al., 2009)**	64/F	India	Ocular trauma	Keratitis	Oral ketoconazole and topical natamycin 5%, econazole 2%, itraconazole 1%,	Corneal perforation, scleral extension
2011 (Zotti et al., 2011)	53/F	Italy	None	Onychomycosis	Oral itraconazole and topical amorolfine	Resolution
2012 (Caira et al., 2012)	66/M	Italy	Acute myeloid leukemia	Invasive pulmonary infection	Voriconazole	Death*
2014 (Tam et al., 2014)	65/M	China	Diabetes mellitus and bronchiectasis	Invasive pulmonary infection	Liposomal amphotericin B and itraconazole	Death
2014 (Tam et al., 2014)	64/M	China	Obsolete pulmonary tuberculosis and bronchiectasis	Chronic cavitary and fibrosing pulmonary and pleural infection	Oral itraconazole	Resolution
2020	73/F	China	Acute myeloid leukemia	Rhinofacial mycosis	Amphotericin B	Death

*Death attributable to underlying disease.**Originally identified as *A. nomiae*; re-identified here as *A. pseudonomiae*.

With regard to PUTH 1342 resistance to antifungals, the MICs/MECs were as follows: AMB, 2 mg/L; voriconazole, 1 mg/L; itraconazole, 0.5 mg/L; posaconazole, 0.25 mg/L; caspofungin, ≤ 0.008 mg/L; and micafungin, ≤ 0.008 mg/L. According to antifungal criteria (EUCAST), *A. flavus* and *A. nomiae* are resistant to AMB and voriconazole, while other *Aspergilli* are susceptible. Therefore, PUTH 1342 was least susceptible to AMB, and most susceptible to the echinocandins (caspofungin and micafungin). The azoles appeared intermediate in their impact on PUTH 1342.

In a comprehensive literature search, we found five published cases of *A. nomiae* infection in humans (Manikandan et al., 2009; Zotti et al., 2011; Caira et al., 2012; Tam et al., 2014), two of which reported incidences of AMB (or any antifungal) resistance. The essential data from patients, including the present case, are summarized in Table 2.

## Discussion

*Aspergillus flavus* is a relatively frequent cause of invasive sinusitis with a global distribution (Webb and Vikram, 2010), particularly in arid climate zones (Taj-Aldeen et al., 2003). Most patients are otherwise healthy, the sinusitis expressing nasal polyps and opacity of the sinus are evident upon CT scan (Taj-Aldeen et al., 2003). With underlying diseases, particularly those impairing the innate immune system, the disorder may become fulminant with potential extension into the brain (Ellis et al., 2002).

The present case was initiated with a rhinofacial ulcer by *A. nomiae*, a species closely related to *A. flavus* and also a member of section *Flavi*. Our findings showed that PUTH 1342 was misidentified, as it was morphologically similar to *A. flavus.* To our knowledge, this is the first report of a rhinofacial mycosis due to *A. nomiae*. Human infections due to *A. nomiae* seem to be rare. To date, infection in only five patients, including the present case, have been confirmed worldwide. Three of these cases were reported in China and two in Italy. The *A. nomiae* isolate from a corneal infiltrate in India (Manikandan et al., 2009) clustered more closely with *Aspergillus pseudonomiae* in our phylogenetic analysis (Figure 5), and this isolate is therefore here re-identified as such. *A. pseudonomiae* is phylogenetically and phenotypically closely related to *A. nomiae*, and was first described 2 years after the reported case (Table 2) (Manikandan et al., 2009). *A. nomiae* is another sibling of *A. flavus* and has repeatedly been misidentified with that species (Tam et al., 2014). Therefore, it may be significantly more common in aspergillosis infections than previously reported. *A. flavus* is susceptible to AMB. For example, Ellis et al. (2002) reported successful application of liposomal AMB in a case of a rhinocerebral infection by *A. flavus*. However, in the present case, AMB proved ineffective against PUTH 1342 and the strain appeared resistant *in vitro*. The limited information available (Manikandan et al., 2009; Caira et al., 2012) indicates that *A. nomiae* may be a species with enhanced resistance to AMB. Notably, angioinvasion was observed in our 73-year-old patient, which was characteristic of rhino-orbital cerebral mycosis and might have played an important role in her cerebral hemorrhage (Li and de Hoog, 2009; Li et al., 2014).

de Hoog et al. (2019), summarizing published antifungal studies against *A. flavus*, noted that for most compounds it showed a wide resistance profile, ranging from totally susceptible to totally resistant. Perhaps this variability is due to misidentification of resistant siblings of *A. flavus*, such as *A. nomiae* (Tam et al., 2014). Retrospective identification and susceptibility testing is required to resolve this uncertainty. The accurate diagnosis of *A. nomiae* infection requires close examination of micromorphology or identification with Maldi-tof (Tam et al., 2014), and unambiguous diagnostics through multilocus sequencing typing (Tam et al., 2014). Serum GM and G tests are widely used for the diagnosis of *Aspergillus* infection, but these tests are non-species-specific and sometimes insensitive (Boch et al., 2018). Although the serum G test was negative, the serum GM test in the case report was positive (1.25 μg/L > 0.5 μg/L) and might have led to certainty of *Aspergillus* infection.

In the limited number of reported human infection cases involving *A. nomiae* published to date, risk factors for fulminant disease included myeloid leukemia (Caira et al., 2012), trauma (Manikandan et al., 2009), diabetes mellitus (Tam et al., 2014), bronchiectasis (Tam et al., 2014), and *Mycobacterium tuberculosis* infection (Tam et al., 2014). Our patient had been diagnosed with acute myeloid leukemia, and during that first year received chemotherapy accordingly. Onset of the nasal necrosis occurred 10 months post-diagnosis during the course of chemotherapy. Reported infections known to involve *A. nomiae* were pulmonary (Caira et al., 2012), ocular (Manikandan et al., 2009), or involving the nails (Zotti et al., 2011). Half of the reported cases (3/6) were pulmonary, including two invasive infections and one chronic pulmonary and pleural infection (Caira et al., 2012). Our case showed rhinofacial involvement. Vascular invasion is an important feature of rhino-cerebral mycosis by Mucorales (Li and Lun, 2012; Li et al., 2014) and has been observed in rhino-orbital aspergillosis (Ben-Ami et al., 2009; Huang and Gui, 2019). Whether or not the lethal cerebral hemorrhage was induced by the angiodestruction of the fungus could not be determined; angioinvasive and angiodestructive characteristics have been observed biopsied in the rhinofacial area. Angioinvasion leading to cerebral destruction has played a role in the sudden death of immunocompromised patients before (Li and de Hoog, 2009; Li et al., 2014), as well as in *A. fumigatus*-triggered thrombosis, hypoxia, and proinflammatory cytokine release (Ben-Ami et al., 2009).

The *A. nomiae* isolate in our patient was resistant to AMB (S ≤ / > R = 1/1) according to the latest drug resistance criteria (Arendrup et al., 2020). This resistance permitted angioinvasion by the fungus, which led to treatment failure. This empirical treatment was chosen on the basis of clinical features of the rhinofacial mycosis, which is mostly caused by members of Mucorales that are generally susceptible to AMB. For invasive *Aspergillus* infection, voriconazole is typically recommended as the first-line drug (Vermeulen et al., 2013; Hagiwara et al., 2016). Limited published data on *A. nomiae* indicate insusceptibility to this compound (Manikandan et al., 2009; Caira et al., 2012). In this study, we found PUTH 1342 to be fairly resistant to voriconazole since it required a higher dose for fungal inhibition. Voriconazole therefore seems to be inappropriate as an initial therapy for *A. nomiae* infections. Correspondingly, due to the low values of *in vitro* drug sensitivity tests, caspofungin (≤0.008 mg/L) and micafungin (≤0.008 mg/L) would be good for first-line antifungals. At the same time, our case reflects the importance of developing a definitive antifungal criterion (EUCAST) for *A. flavus* and *A. nomiae*, especially in clinical practice.

*Aspergillus nomiae* strains are able to produce both series B and G aflatoxins (Ehrlich et al., 2004), which are notorious for hepatocellular carcinoma development, lung adenocarcinoma, and chronic inflammatory changes (Cha and Dematteo, 2005; Liu et al., 2015). Our patient’s white blood cell counts increased sharply after respiratory tract infection, and subsequently she was diagnosed with leukemia. Nasal necrosis by *A. nomiae* developed during chemotherapy. We are not sure whether the aflatoxins contributed to the malignancy. Given that the fungus had infiltrated her nasal cavity, perhaps she was swallowing mucus and fungal spores. Ingestion of aflatoxins is where the problems arise. They end up in the liver and cannot be removed so they accumulate and may lead to hepatocarcinoma. Depending on the concentration of aflatoxins, they may have contributed to her death as aflatoxicosis. The poor physical state of most patients with invasive fungal infection limits the efficiency of antifungal drugs. Of the patients with invasive infection due to *A. nomiae*, including the present one, all died. In contrast, patients with non-invasive infections, including chronic pulmonary infection, keratitis, and onychomycosis, have all been cured with antifungal treatment.

We report this case to increase the public awareness of *A. nomiae* infections. Given that the morphological characteristics of *A. nomiae* are similar to those of *A. flavus* and molecular identification of *Aspergillus* is not routinely performed in clinical mycology laboratories, *A. nomiae* infections are suspected to be underreported. Rhinofacial mycoses involving *A. nomiae* might be fatal if they cannot be identified in a clinical lab providing appropriate treatment and if they have reduced susceptibility to AMB.

## Data Availability Statement

The datasets presented in this study can be found in online repositories. The names of the repository/repositories and accession number(s) can be found in the article/supplementary material.

## Ethics Statement

This research has been approved by Peking University Third Hospital IRB; approval #00006761-2015025. Written informed consent was obtained from the next of kin for the publication of any potentially identifiable images or data included in this article.

## Author Contributions

DL diagnosed the patient, designed the experiments, and wrote partial manuscript. YZ did the experiment and wrote the manuscript. TS assisted in experiment. JH made the tree. GH reviewed the manuscript. All authors contributed to the article and approved the submitted version.

## Conflict of Interest

The authors declare that the research was conducted in the absence of any commercial or financial relationships that could be construed as a potential conflict of interest.

## Footnotes

1 http://www.ncbi.nlm.nih.gov
